# Severe tissue complications in patients of *Bothrops* snakebite at a tertiary health unit in the Brazilian Amazon: clinical characteristics and associated factors

**DOI:** 10.1590/0037-8682-0374-2020

**Published:** 2021-02-26

**Authors:** Flavio Souza Silva, Hiochelson Najibe Santos Ibiapina, Juliana Costa Ferreira Neves, Kerolaine Fonseca Coelho, Fabiane Bianca Albuquerque Barbosa, Marcus Vinicius Guimarães Lacerda, Jacqueline Almeida Gonçalves Sachett, Adriana Malheiro, Wuelton Marcelo Monteiro, Allyson Guimarães Costa

**Affiliations:** 1 Fundação de Medicina Tropical Dr. Heitor Vieira Dourado, Instituto de Pesquisa Clínica Carlos Borborema, Manaus, AM, Brasil.; 2 Universidade do Estado do Amazonas, Programa de Pós-Graduação em Medicina Tropical, Manaus, AM, Brasil.; 3 Fundação Oswaldo Cruz, Instituto de Pesquisas Leônidas e Maria Deane, Manaus, AM, Brasil.; 4 Fundação Alfredo da Matta, Diretoria de Ensino e Pesquisa, Manaus, AM, Brasil.; 5 Fundação Hospitalar de Hematologia e Hemoterapia do Amazonas, Diretoria de Ensino e Pesquisa, Manaus, AM, Brasil.; 6 Universidade Federal do Amazonas, Programa de Pós-Graduação em Imunologia Básica e Aplicada, Manaus, AM, Brasil.; 7 Universidade do Estado do Amazonas, Programa de Pós-Graduação em Ciências Aplicadas à Hematologia, Manaus, AM, Brasil.

**Keywords:** *Bothrops* snakebites, Envenomation, Severe tissue complication, Associated factors, Brazilian Amazon

## Abstract

**INTRODUCTION:**

Snakebites in the Brazilian Amazon are caused mostly by snakes from the *Bothrops* genus and envenomated patients may suffer from tissue complications.

**METHODS:**

This study aimed to identify risk factors for severe tissue complications (STC) in patients with *Bothrops* snakebite in the Amazonas state, Brazil.

**RESULTS:**

Snakebites that were classified as severe and affected female patients with comorbidities presented greater risks of developing STCs. In addition, hospitalizations of patients with STC exceeded 5 days.

**CONCLUSIONS:**

Clinical and epidemiological characteristics can prove essential for assessing the evolution of STC and clinical prognosis of patients with *Bothrops* snakebites.

Snakebites occur frequently worldwide, mainly in the tropical and subtropical countries of Africa, Asia, America, and Oceania. They are considered a severe and neglected public health problem that affects millions of people worldwide annually, especially in countries with high agricultural activity^1^. Snakebites affect productive individuals in communities, such as rural workers, and can cause losses to the economy and financial hardships to families^2^ ^,^ ^3^.

In Brazil and the rest of the Amazon region, *Bothrops* snakes are responsible for about 80%-90% of reported snakebites^4^. *Bothrops* venom contains molecules such as metalloproteases, serinoproteases, phospholipase A2, and L-amino acid oxidases that can cause inflammatory processes, hemostatic disorders, and tissue damage resulting in signs and symptoms characteristic of this type of snakebite^5^.

Tissue injury resulting from *Bothrops* snakebites can present manifestations such as pain, edema, erythema, ecchymosis, and severe complications such as blistering, abscess, necrosis, and compartment syndrome^2^ ^,^ ^6^. Severe tissue complications (STC) can result in permanent sequelae for individuals who develop them, harming them physically and economically^7^. Therefore, it is essential to analyze the frequency of these complications and their sequelae to assess the intensity of the problem and provide clearer information to policy makers and health authorites^8^.

Thus, this study aimed to describe the frequency of STCs caused by *Bothrops* snakebites in patients treated at a referral hospital in the Brazilian Amazon as well as to analyze the risk factors associated with the development of STC.

In the state of Amazonas, Fundação de Medicina Tropical Dr. Heitor Vieira Dourado (FMT-HVD), located in the city of Manaus-AM, is the reference center in the northern region for the treatment of tropical diseases including snakebites, and it attends to patients from both the capital and the municipalities in the interior of the state.

The data of interest for the study were obtained from the iDoctor system database at the FMT-HVD to obtain clinical information regarding patient admissions due to *Bothrops* snakebites that occurred between January 1, 2012, and December 31, 2016.

Epidemiological and clinical data, such as the date of diagnosis, age group, sex, area where the snakebite occurred (rural or urban), anatomical location of the snakebite, length of hospital stay, clinical classification of envenomation (mild, moderate, or severe), administration of antivenom, presence of comorbidities, and STCs were included. The cases were classified into mild, moderate, and severe cases according to the Brazilian Health Ministry guidelines. To identify the risk factors for STCs following *Bothrops* snakebites, a case-control study was performed, in which patients with STCs were termed as cases and those without STCs were termed as controls. 

Statistical analyses were performed using STATA v. 13 (StataCorp LLC, College Station, TX, USA). Odds ratios (OR) and 95% confidence intervals (95% CI) were calculated using a logistic regression analysis. A backward stepwise technique was applied. Variables with p-values less than or equal to 0.2 in the univariate logistic regression were selected for the multivariate model analysis. Goodness-of-fit for the logistic regression model was determined using the Hosmer-Lemeshow test. For the final model, statistical significance was defined as p <0.05.

In the period evaluated in the study, 859 electronic medical records of patients with snakebites were identified. Of these, only 307 were included in the study, as these records were considered complete with respect to their descriptions of the clinical signs and symptoms of the patients’ *Bothrops* envenomation as well as the severe complications presented by the patients and treated at FMT-HVD.

According to the medical records included in the study, the majority of *Bothrops* snakebites mainly affected individuals aged 16 to 45 (57%) years and most were male (79%). The lower limb was the most affected anatomical region (88%). Most snakebites occurred in rural areas (72%), were classified as moderate (63%), and *Bothrops* antivenom was the treatment administered in 95% of the cases. Among the records evaluated in the study, 2% of the patients had comorbidities. Furthermore, the zone of occurrence, anatomical site of the snakebite, and days of hospitalization variables were not described in all the medical records consulted and were analyzed wherever information was available. In addition, 47% individuals remained hospitalized for up to 2 d after hospital admission. Analysis of the 307 records revealed that 19% patients had STCs and that some patients had more than one type of complication. The STCs listed in the records were ecchymosis (8%), abscess (7%), blister (5%), necrosis (2%), and compartment syndrome (1%) (Table 1).

**TABLE 1:** Demographic and clinical characteristics in patients hospitalized due to *Bothrops* snakebites.

Characteristics (Completeness)	n (%)
**Age group in years**	**307 (100)**
0-15	33 (11)
16-45	176 (57)
46-65	72 (24)
>65	26 (8)
**Sex**	**307 (100)**
Male	242 (79)
Female	65 (21)
**Zone of occurrence**	**246 (80)**
Rural	177(72)
Urban	69 (28)
**Anatomical site of the snakebite**	**298 (94)**
Upper limb	34 (12)
Lower limb	254 (88)
**Snakebite classification**	307 (100)
Mild	38 (12)
Moderate	194 (63)
Severe	75 (25)
**Antivenom therapy**	**307 (100)**
*Bothrops* antivenom	292 (95)
*Bothrops/Lachesis* antivenom	15 (5)
**Comorbidities**	**307 (100)**
No	300 (98)
Yes	7 (2)
**Days of hospitalization**	**289 (94)**
1-2	137 (47)
3-5	84 (29)
>5	68 (24)
**Local complication**	**307 (100)**
No	248 (81)
Yes	59 (19)
**Description of the local complication**	**307 (100)**
Bruising	25 (8)
Abscess	20 (7)
Blister	15 (5)
Necrosis	6 (2)
Compartmental Syndrome	4 (1)

Sex, snakebite classification, presence of comorbidities, and length of hospital stay were independently associated with the development of STCs. Female patients and snakebites classified as severe showed a two-fold higher risk (OR: 2.63; p=0.006; OR: 2.40; p=0.008, respectively) of developing STC. In addition, patients with comorbidities (diabetes and hypertension) were 8 times more likely to develop STCs (OR: 8.53; p=0.022). Finally, patients with STCs were at risk of a longer period of hospitalization, which was longer than 5 days (OR: 2.70; p=0.003) (Table 2).

**TABLE 2:** Factors associated with severe tissue complications in patients with *Bothrops* snakebites

Variables	STC		Crude OR	p-value	Adjusted OR	p-value*
	Yes (%)	No (%)	(CI 95%)		(CI 95%)	
**Age group in years**						
0-15	8 (13)	25 (10)	-	1	-	1
16-45	27 (46)	149 (60)	0.57	0.213	-	1
			(0.23-1.39)			
46-65	17 (29)	55 (22)	0.97	0.944	-	1
			(0.37-2.53)			
>65	7 (12)	19 (8)	1.15	0.814	-	1
			(0.35-3.73)			
**Sex**						
Male	39 (66)	203 (82)	-	1	-	1
Female	20 (34)	45 (18)	2.31	0.0009	2.63	0.006
			(1.23-4.33)		(1.32-5.26)	
**Zone of occurrence**						
Rural	31 (66)	146 (73)	-	1	-	1
Urban	16 (34)	53 (27)	1.42	0.311	-	1
			(0.72-2.81)			
**Anatomical site of the snakebite**						
Upper limb	8 (14)	26 (11)	-	1	-	1
Lower limb	49 (86)	204 (89)	0.78	0.569	-	1
			(0.33-1.82)			
**Snakebite classification**						
Mild	2 (3)	36 (14)	-	1	-	1
Moderate	34 (58)	160 (65)	3.82	0.074	3.31	0.118
			(0.88-16.56)		(0.74-14.84)	
Severe	23 (39)	52 (21)	7.96	0.007	2.40	0.008
			(1.77-35.90)		(1.26-4.58)	
**Antivenom therapy**						
*Bothrops* antivenom	54 (92)	238 (96)	-	1	-	1
*Bothrops/Lachesis* antivenom	5 (8)	10 (4)	2.20	0.164	1.64	0.480
			(0.72-6.71)		(0.42-6.43)	
**Comorbidities**						
No	55 (93)	245 (99)	-	1	-	1
Yes	4 (7)	3 (1)	5.93	0.022	8.53	0.022
			(1.29-27.30)		(1.37-53.14)	
**Days of hospitalization**						
1-2	17 (31)	120 (51)	-	1	-	1
3-5	17 (31)	67 (29)	1.79	0.121	1.79	0.142
			(0.86-3.74)		(0.82-3.89)	
>5	21 (38)	47 (20)	3.15	0.002	2.70	0.003
			(1.53-6.50)		(1.39-5.23)	

*OR: odds ratio; **CI:** confidence interval; **Crude:** univariate logistic regression; **Adjusted:** multivariate logistic regression.

Epidemiological data referring to age, sex, area of snakebite occurrence, affected limb, and classification of the snakebite are consistent with previous studies conducted in the Amazon region^2^. In addition, medical records with missing information were also observed in tertiary health units, impacting the completeness of the data to be analyzed, as seen in other studies^4^ ^,^ ^7^. These characteristics are mainly related to the influence of the development of socioeconomic activities in rural areas, especially agricultural and extractive activities. Thus, individuals in the productive age group who are affected end up suffering economically because of the non-development of socioeconomic activities. This occurs in the recovery period when the clinical and physical conditions caused by the envenomation require medical follow-up and because of the resulting sequelae^2^ ^,^ ^9^.

Antivenom serotherapy was performed with *Bothrops* antivenom (95%) and *Bothrops/Lachesis* antivenom (5%) **(** Table 1 **)**. Although we did not observe this in our study, conditions of delay or absence of antivenom serotherapy may favor the occurrence of local complications in the clinical picture of injured patients in the Amazon region^10^. The difficulty of traveling from the rural area to the capital due to the great territorial expanse of the region and the predominance of river transport in the interior of the state of Amazonas resulting in journeys lasting several days are some obstacles involved^9^. In addition, empirical therapeutic measures such as the use of a tourniquet on the affected limb can also lead to the development of tissue complications^6^ ^,^ ^11^.

In a series of snakebite cases, it was confirmed that envenomation inflicted by adult snakes can cause more severe tissue inflammatory effects whereas that inflicted by juvenile snakes results in venom-induced coagulopathy more frequently^12^. Despite this, persistent bleeding at the site of the bite is not very frequent^13^. Cases of amputation and fasciotomy have been described in the treatment of tissue complications such as necrosis and compartment syndrome, respectively^14^.

Furthermore, blisters can act as a gateway for pathogenic microorganisms that can cause secondary infections^7^. The most common complications (ecchymosis, abscess, and blister) can progress to more severe forms of STC. Compartment syndrome is considered the most severe, and although it has an uncommon occurrence, it can result in tissue necrosis, ischemia, and neuropathy^15^. In addition, depending on the severity, necrosis can trigger functional sequelae and, consequently, lead to amputation of the affected limb^6^. 

Specific medical procedures are required in cases of envenomation by *Bothrops* sp. if the presence of tissue complications, such as ecchymosis, abscess, blisters, necrosis, and compartment syndrome, are evident^2^ ^,^ ^7^ ^,^ ^13^ ^,^ ^15^. For abscesses, drainage surgery is necessary^2^. In cases of compartment syndrome, the patient can usually be treated by fasciotomy since in this complication there may be ischemia, tissue necrosis, and neuropathy that contribute to its worsening^6^ ^,^ ^13^ ^,^ ^15^. In addition, tissue necrosis almost always requires a surgical procedure for removal of both necrotic and devitalized tissues and tissue repair or reconstitution. When this complication is greatly aggravated, it can lead to functional deficits^2^ ^,^ ^15^. Moreover, patients with STCs had longer hospitalization periods since they require specific hospital care and, consequently, costs for the health center for the treatment of snakebites are greater. 

*Bothrops* snakebite in patients of the female gender, classified as severe, and with the presence of comorbidities is the profile most indicative of the risk of tissue complications among the cases reported in the Amazon region. Thus, it is noted that simple epidemiological and clinical observations can prove to be important information since they can be used to assess patient prognosis and the possibility of the development of complications after envenomation.
